# Identity change and the transition to university: Implications for cortisol awakening response, psychological well‐being and academic performance

**DOI:** 10.1111/aphw.12608

**Published:** 2024-10-18

**Authors:** Siobhán M. Griffin, Alžběta Lebedová, Tegan Cruwys, Grace McMahon, Aoife Marie Foran, Magdalena Skrodzka, Stephen Gallagher, Annie T. Ginty, Orla T. Muldoon

**Affiliations:** ^1^ Centre for Social Issues Research, Department of Psychology University of Limerick Limerick Ireland; ^2^ Department of Psychology and Neuroscience Baylor University Waco Texas USA; ^3^ School of Medicine and Psychology The Australian National University Canberra Australia; ^4^ School of Psychology The University of Queensland Brisbane Queensland Australia; ^5^ School of Psychology Queen's University Belfast Belfast Northern Ireland

**Keywords:** cortisol awakening response (CAR), depression, social group memberships, social identity change, social identity model of identity change (SIMIC), well‐being

## Abstract

The social identity model of identity change (SIMIC) posits that social group memberships protect well‐being during transitional periods, such as the transition to university, via two pathways — maintaining previously held social group memberships (social identity continuity) and gaining new social group memberships (social identity gain). Breaking new ground, this study investigates how these processes can influence an important biomarker of stress — cortisol awakening response (CAR). A total of 153 first year undergraduate students (69.3% female) completed measures (group memberships, depression, life satisfaction) at the beginning of the academic year (October, time 1; T1), of which 67 provided a saliva sample for CAR assessment. Seventy‐nine students completed the time 2 (February, T2) measures 4 months later (41 provided saliva). Academic performance was assessed objectively through end‐of‐academic year university grade data (June, T3). At T1, students who maintained and gained social group memberships reported lower depressive symptoms and greater life satisfaction. Across the academic year, social identity gain was associated with a larger post‐awakening cortisol response at T2, indicative of a better ability to cope with stress. Thus, gaining new social group memberships during the transition to university was associated with a better ability to cope with stress.

## INTRODUCTION

A successful transition to university (academic, social and emotional adjustment; e.g. Baker & Siryk, 1984) is important for student's well‐being, academic performance and likelihood to complete their degree (for reviews, see Credé & Niehorster, 2012; Sahão & Kienen, 2021; van der Zanden et al., 2018). It is imperative to support students during this transition as it coincides with a high‐risk period of developing mental health difficulties, with approximately 75% of serious mental health conditions first occurring between 15 and 25 years (Kessler et al., 2005). High rates of mental health difficulties in university student populations are well documented (for reviews see; de Paula et al., 2020; Sheldon et al., 2021). For instance, the World Health Organization found 35% of first year students (>13,000 students, eight countries) screened positive for common mental health disorders (Auerbach et al., 2018). Students face a number of stressors, including pressures to excel academically, increased independence and responsibility, financial pressures (including juggling part‐time working) and coping in a new social environment — often away from their family and established friendship groups (Acharya et al., 2018; Szabo & Marian, 2017; Worsley et al., 2021). For some, it can lead to the loss of previously held social groups and associated social identities, which has negative implications for psychological health (Oswald & Clark, 2003; Scanlon et al., 2007). However, university also offers opportunities to explore different identities and activities, fostering the creation of new social groups. These social group memberships are posited, in line with the social cure literature, to contribute to people's sense of self (Tajfel, 1982) and affect health. Previous empirical research has demonstrated the importance of social group memberships for adjustment to university in terms of well‐being and academic performance (Cruwys et al., 2021; Foran et al., 2023; Praharso et al., 2017). We build on this work, by examining if social group memberships can also influence an established stress biomarker — cortisol awakening response (CAR).

## GROUP MEMBERSHIPS AND PSYCHOLOGICAL WELL‐BEING

The social identity approach (Tajfel, 1982; Turner et al., 1987), when applied to health, places an emphasis on how people define themselves through membership of various social groups. People internalize these group memberships as part of their sense of self (i.e. develop a sense of social identity as a group member), and this has important implications for health (C. Haslam et al., 2018; Jetten et al., 2015; Kearns et al., 2018). These group memberships offer *social identity resources* that people can draw on, including social support, solidarity and a sense of belonging (Kinsella et al., 2023; Muldoon, Walsh, et al., 2019). These resources, irrespective of the group membership from which they are drawn, have been shown to mitigate the impact of stress and promote mental health and resilience (Jetten et al., 2015; Muldoon, Walsh, et al., 2019; White et al., 2021). The more group memberships a person has, the greater the benefits (e.g. lower depressive symptoms), as it means more psychological resources to draw upon promoting resilience to the effects of stress — particularly during life transitions (e.g. Cruwys et al., 2013, 2014, 2023; Haslam et al., 2021, 2022; Iyer et al., 2009; Jetten et al., 2014; Jones et al., 2012; Kim et al., 2023; Kinsella et al., 2018; Walsh et al., 2015).

**FIGURE 1:**
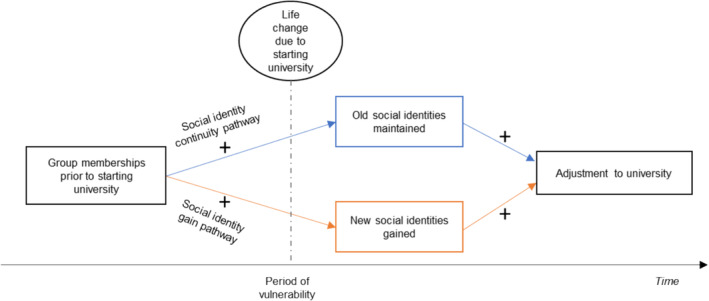
SIMIC model depicting pathways through which social group memberships can influence adjustment to university (adapted from; Haslam et al., 2018, p. 238).

The social identity model of identity change (SIMIC; Haslam et al., 2018) proposes two processes through which social group memberships help people navigate life transitions: the social identity continuity pathway and the social identity gain pathway, as depicted in Figure 1. The latter arises as life changes often offer opportunities to join new social groups and gain new social identities that flow from them. When old identities are lost during a transition, new group memberships can help buffer this loss and provide a new sense of self (Muldoon, Haslam, et al., 2019). Developing new group memberships has been shown to promote better psychological well‐being after moving to university (Iyer et al., 2009; Ng et al., 2018). Likewise, maintaining social group memberships that were held prior to a transition (social identity continuity) predicts better well‐being, for instance in young athletes transitioning into a professional career pathway (Rees et al., 2022) and international students transitioning to university (Cruwys et al., 2021; Praharso et al., 2017). However, successful transition to a university environment also requires effective engagement with the intellectual challenges of university itself: reflected in academic performance. Greater academic performance in the first year has been linked with increased likelihood to graduate from ones degree (Gershenfeld et al., 2016; Westrick et al., 2023), and overall performance is associated with better employment and salary opportunities (Cable & Judge, 1997; Roth & Clarke, 1998). Previous research has found that SIMIC processes (particularly, identity continuity) predict stronger academic performance in university students (e.g. Cruwys et al., 2021) due to the psychological resources offered by group memberships. Whilst this research demonstrates social group memberships have direct effects on performance, it is likely that SIMIC pathways can also affect performance indirectly via psychological well‐being (e.g. lower depression, lower stress and greater life satisfaction), considering the links between well‐being and academic performance (e.g. Antaramian, 2017; Mihăilescu et al., 2016). Whilst the relationship between SIMIC pathways and subjective well‐being is well documented, there is little evidence of a relationship between these pathways and a biomarker of stress.

## GROUP MEMBERSHIPS AND PHYSICAL HEALTH

Social identity processes and group memberships are inherently linked to stress responses. Specifically, the integrated social identity model of stress (Haslam & Reicher, 2006) proposes that the psychological and material resources offered by shared social group memberships can be drawn on during times of stress (Haslam et al., 2004; Haslam & Reicher, 2006) feeding into stress appraisals (i.e. the perceived ability to cope with situational demands) and subsequent physiological responses to stress. Speaking to this, experimental work has shown that manipulating a sense of shared social identity or group status during a laboratory stress task affects physiological stress responses, such as blood pressure (Gallagher et al., 2014; McMahon et al., 2024; Scheepers, 2009), as well as cortisol‐mediated responses to stress (Häusser et al., 2012). Likewise, other empirical evidence demonstrates a link between group memberships and longer‐term physical health outcomes (e.g. Putnam, 2000; Steffens et al., 2016). Perhaps most compelling is evidence that if a person does not belong to a group but joins one, they reduce their mortality risk in the subsequent year by approximately 50% (Putnam, 2000). Indeed, maintaining social group memberships at a key transitional stage (retirement) was associated with lower mortality risk (Steffens et al., 2016). Findings such as these, along with work linking social bonds with other biomarkers such as oxytocin and opioids (e.g. Inagaki et al., 2020; Pearce et al., 2017), suggest that maintaining and/or gaining groups affects biomarkers of stress during times of transition, which over time might have implications for physical health.

In extending this work, we examine CAR, which is the increase in cortisol observed after awakening. It is a neuroendocrine manifestation of the HPA axis to face anticipated stress (Chida & Steptoe, 2009). Cortisol increases sharply during the first 30–45 min after waking, peaks and then declines throughout the day (Pruessner et al., 1997). In healthy adults, an increase of 50–156% in cortisol is typically seen after waking (Clow et al., 2004; Pruessner et al., 1997). These increases are a normal adaptive neuroendocrine process. CAR is a well‐established non‐invasive biomarker of stress (for a review see; Chida & Steptoe, 2009), which correlates well with other methods of assessing cortisol (e.g. blood; Kim et al., 2020; Köksal, 2022). Larger post‐awakening cortisol responses signal an ability to better cope with daily stressors (Powell & Schlotz, 2012) and are associated with lower worry and greater resilience (Lai et al., 2020; O'Connor et al., 2021). Whilst a lower (blunted) increase in CAR is seen in individuals experiencing chronic stress (Gallagher et al., 2016; Giglberger et al., 2022) or with subclinical depression (Dedovic et al., 2010), mixed findings have been noted (e.g. Steptoe & Serwinski, 2016). It is well established that maladaptive physiological stress responses have long‐term implications for physical health including higher incidence of cardiovascular disease (Chida & Steptoe, 2010; Walther & Wirtz, 2023). Indeed, people with larger CARs are at lower risk of adverse physical health outcomes over time, such as cardiac events and mortality (e.g. Karl et al., 2022; Ronaldson et al., 2015), and less likely to report health problems or a chronic disease (Gartland et al., 2014; Kudielka & Kirschbaum, 2003).

## THE PRESENT RESEARCH

This research aimed to replicate previous research demonstrating that SIMIC pathways are associated with better well‐being and academic performance and extend this work by exploring if social identity gain and continuity are associated with a stress biomarker, CAR, in first‐year undergraduate students transitioning to university. Furthermore, we explored if SIMIC pathways would be indirectly linked with academic performance via life satisfaction, CAR and depression. Students completed measures twice, 4 months apart, during the third and fourth weeks of their first semester in October (time 1; T1) and second semester in February (time 2; T2). End‐of‐year grade data were accessed to provide information on academic performance in June (time 3; T3). The study spanned 8 months, from T1 to T3.

We hypothesized that the social identity gain and continuity pathways would be associated with lower depressive symptoms, greater life satisfaction and a larger increase in CAR at T1 (cross‐sectionally; H1) and T2 (longitudinally; H2), as well as better academic performance (H3). We also explored, based on the links between well‐being and academic performance, if gain and continuity pathways could have indirect effects on academic performance via well‐being and CAR (H4). In this model, respective T1 measures are controlled for, as well as pre‐university group memberships as these predict a greater likelihood of maintaining pre‐transition groups and joining new groups (e.g. Iyer et al., 2009).

## METHODS

### Design and sample

A longitudinal design was used, as described previously. The study was open to all first‐year undergraduates at an Irish university, and students could choose to complete only the questionnaires or to also provide saliva samples. This option was provided to maximize participation for the subjective outcomes as past research documents low uptake for saliva collection (e.g. response rates of 42%–54%; Dykema et al., 2017; Kilpatrick et al., 2007) and due to ethical considerations (all aspects of the study were voluntary).

Ethical approval was obtained from the institutional research ethics committee. Participation was voluntary, participants provided informed consent and could withdraw from the study at any time. Participants who provided saliva samples were offered a €10 voucher for costs incurred in travelling to campus to return their cortisol packs.

### Participants

At time 1 (T1), 153 first year undergraduate students aged 18–20 (*M* = 18.52, *SD* = 0.61 years) participated (106 females, 44 males, one person identified as gender fluid and two people selected other/prefer not to say). Four months later, 79 of these participants completed T2 measures. A subsample of participants chose to provide saliva samples and met the eligibility check to do so (i.e. no current illness such as a cold/flu, non‐smoker, not pregnant, not taking glucocorticoids or immunosuppressants, no chronic immune condition, heart‐related illness or oral/periodontal disease; consistent with best practice guidelines; Stalder et al., 2016); a total of 67 and 41 participants provided samples at T1 and T2, respectively. After assaying, this resulted in CAR data from 58 participants at T1 (42 females) and 38 participants at T2 (29 females). CAR was unable to be calculated for some participants (T1, *n* = 9; T2, *n* = 3), due to insufficient saliva volumes or due to physiologically implausible values (e.g. 0 or an extremely high value within a day); this issue is commonly seen in CAR research (e.g. Giglberger et al., 2022; O'Connor et al., 2021). End of semester grade data was retrieved for the 114 participants who consented this. Full demographics are presented in Table S1, and Figure S1 displays a study flow chart (with attrition rates).

A priori power analyses were conducted using the semPower statistical package (Moshagen & Erdfelder, 2015) for *R* statistical software (R Core Team, 2021). Past research examining SIMIC pathways in students transitioning to university reported small‐to‐medium effects (Cruwys et al., 2021) and large effects (Praharso et al., 2017). To detect misspecifications of the proposed model (using an alpha of .05 and power of .80), a minimum of 85 participants was needed to detect a small effect and 39 to detect a medium effect. As such, the present study had the power to detect medium‐to‐large effects.

### Recruitment

The study was advertised to all first‐year students on departmental social media channels, the psychology research participation system (SONA) where students could complete the study for course credits, via posters around campus and via mass email to all first‐year students in the university. These contained a QR code to a Qualtrics survey where participants read an information sheet, indicated which elements of the study they could take part in (e.g. all parts or questionnaire only) and provided their email address for further information. Participants were then emailed a Qualtrics survey link to provide informed consent, complete the questionnaires and if applicable either book a time to collect their cortisol kit or have it posted to their address.

### Measures

#### Social group memberships

The Exeter Identity Transition Scales (EXITS; Haslam et al., 2008) assessed feelings of subjective identification (i.e. a sense of belonging, connection and support) with social group memberships. This scale has been validated in a range of populations (e.g. Craig et al., 2022; Griffin et al., 2022; Kim et al., 2023) and comprises of three subscales (four items each) assessing connectedness with the following: multiple groups *prior* to starting university (e.g. ‘Before I became a university student, I joined in the activities of lots of different groups’), group *maintained* since starting university and new groups formed *after* starting university. Items from each subscale mirrored each other. Cronbach's alphas for this scale, and others, at each timepoint, are presented in Table S2.

#### Depressive symptoms

Twenty items of the 21‐item Beck Depressive Inventory‐II (BDI‐II; Beck et al., 1996) assessed depressive symptomology (the suicidal thoughts item was not presented). This scale is well‐validated (e.g. Wang & Gorenstein, 2013).

#### Satisfaction with life

The five‐item Satisfaction with Life Scale (SWL; Diener et al., 1985) assessed life satisfaction and has good psychometric properties (e.g. Hinz et al., 2018).

#### Cortisol assessment and analysis

Participants received a cortisol kit that we compiled, containing instructions, a record sheet to indicate sample timings and eight labelled Salivettes sampling tubes (Sarstedt Ltd.). Participants provided four samples per day, for 2 days, at T1 and T2, in line with expert consensus guidelines (Stalder et al., 2016); see the “Procedure” section for full details.

Samples were thawed and centrifuged at 3000 RPM for 10 min using a Hettich Rotofix 32A Centrifuge using a Swing‐Out Rotor, 4‐Place (cat no. 1624) with a maximum RPM of 4000 or relative centrifugal force (RCF) of 2451. Samples were assayed using commercially available enzyme‐linked immunosorbent assay (ELISA) kits for high‐sensitivity cortisol (DRG Diagnostics, Marburg, Germany). The optical density of the samples was read using a Biotek ELX800 (BioTek, Vermont, USA) plate reader and Gen5 software (BioTek, Vermont, USA) at 450 nm. Each sample was tested in duplicate, with the mean value used in the statistical analyses. To control for differences in participants' daily activity, saliva samples were collected across two consecutive days and a mean value was computed (ng/mL). There were no significant differences in cortisol across these 2 days (T1; *p* = 0.143, T2; *p* = .138). Intra‐assay % coefficient of variation (%CV) for cortisol was 5.87%.

#### Academic performance

This was obtained from the official university grading system at the end of the academic year, indicated by participant's quality credit average (QCA) averaged across the first and second semester. A score of 4.0 represents a perfect score.

### Procedure

Students were emailed a Qualtrics link to complete the questionnaires. Students who agreed to CAR assessment and met the inclusion criteria collected their cortisol kit on‐campus or received it by post. Instructions for saliva collection and storage were provided in print, via email and via an instructional video. Participants were asked to tip the cotton swab into their mouth and let saliva collect naturally for a minimum of 2 min until the swab was saturated, then tip it back into the container and seal it. Participants were told to not consume food or liquid (except for water) and not to brush their teeth during this time. Participants provided eight saliva samples across two consecutive normal weekdays (four samples per day; immediately upon wakening and then at 15 min, 30 min and 45 min post‐awakening), consistent with guidelines (Dockray et al., 2008; Stalder et al., 2016, 2022). Salivettes were organised by day and time and colour coded to match the record sheet. On the record sheet, each time period (e.g. +15 min) had a corresponding hyperlink to a google survey where participants uploaded a photo of each completed sample. This acted as an adherence check, as each upload was timestamped by the survey (Stalder et al., 2022). Participants stored the samples in their own refrigerator and returned them within 2–3 days, where they were stored in our laboratory at −20°C until assay. Although cortisol in saliva is stable at room temperature for at least 4 weeks (Kirschbaum & Hellhammer, 2007), transportation of the samples to campus was likely to be less than a few hours. If participants failed to adhere to the protocol, they could request new cortisol packs; this happened to two participants (one provided samples at the incorrect times, the other dropped a sample on the floor), new kits were provided and this data was used. The procedure was repeated at T2. End‐of‐semester grade data was retrieved at T3 (June) after students received their exam results.

### Data analysis

To examine if social identity gain and continuity at T1 were associated with well‐being and CAR at T1, bivariate correlations were conducted (H1). To test SIMIC predictions over time (H2–H4), we conducted path analysis using AMOS to test the hypotheses simultaneously. To maximize power, full information maximum likelihood (FIML) was used which retains all available observations from participants rather than excluding listwise those who were not retained at subsequent timepoints (Allison, 2012). The predictors were change in group memberships maintained post‐transition (T2) and change in new group memberships gained post‐transition (T2). The outcome variables were change in depressive symptoms (T2), life satisfaction (T2), CAR (T2) and academic performance (T3). Time 1 measures of these data were all entered in the model as predictors of their T2 equivalent, including depressive symptoms (T1), life satisfaction (T1), CAR (T1), new group memberships (T1) and maintained group memberships (T1) to control for these variables. Pre‐university group memberships (T0) were entered as a predictor of maintained and new groups (T2) to control for this. Error terms were specified for the endogenous variables. All T1 variables were allowed to covary, as well as maintained and new group memberships at T2 as these are expected to occur in concert and be correlated (see also Cruwys et al., 2021). Time 2 health variables (depression, life satisfaction and CAR) were allowed to covary. Means and standard deviations are presented in Table S1. Path analysis was chosen over a full SEM as the focus of this research, a priori, was on the structural component rather than the measurement component (Deng & Yuan, 2023; Grapentine, 2000), and we did not hypothesize differences in the internal structure of the latent variables due to these being measures being well‐validated in the literature (Haslam et al., 2008; Hinz et al., 2018; Wang & Gorenstein, 2013). However, it is worth noting that path models are limited in their ability to handle measurement errors relating to the latent constructs (Bollen & Lennox, 1991; Bollen & Pearl, 2013). Whilst we lacked sufficient power to run a full SEM, we report the results of a full SEM using multiple imputation in the Supplementary Materials. However, we advise caution in interpreting these results as data imputation with a large proportion of missing data is not advised (Lee & Huber, 2021).

Model fit was tested using AMOS with the maximum likelihood estimation.^1^ Model fit was assessed using five indices from the three primary categories of fit: absolute fit indices (*χ* ^2^, Akaike Information Criterion [AIC] and the recommended AIC_c_ for smaller samples [for a discussion, see Burnham & Anderson, 2004]), root mean square error of approximation (RMSEA), a relative fit index (Bentler‐Bonett Normed Fit Index [NFI]) and a non‐centrality‐based index (comparative fit index [CFI]), as recommended by Hu and Bentler (1999). Models with lower RMSEA and higher CFI and NFI are thought to be better fitting. Whilst there are no strict cut‐offs for evaluating fit indices, generally a RMSEA value between .05 and .08 suggests fair fit (>.05 suggests close fit), and NFI and CFI values higher than .90 indicate good fit (Marsh & Hau, 1996). We report AIC and AIC_c_ values, with lower values indicative of a more parsimonious model (Aho et al., 2014),^2^ but note as these are relative values, they should be compared to the alternative models presented in the Supplementary Materials.

Previous research has documented cortisol values that are skewed and recommended using log_10_ transformations (Gallagher et al., 2016; Giglberger et al., 2022; O'Connor et al., 2021). In this study, the mean cortisol values across both days were positively skewed and log_10_ transformations were conducted prior to analyses. To calculate CAR, area under the curve with respect to increase (AUC_i_) was computed on non‐transformed values (Pruessner et al., 2003).^3^

## RESULTS

### Testing SIMIC pathways

Bivariate correlations demonstrated that the maintenance of pre‐university group memberships was associated with lower depressive symptoms (*p* < .001) and greater life satisfaction (*p* = .003), but unrelated to CAR (*p* = .606) at T1. New group memberships at T1 were also associated with lower depressive symptoms (*p* = .017) and greater life satisfaction (*p* = .001), but not CAR (*p* = .971). As such, H1 was only partially supported. Bivariate correlations between all variables are presented in Table S5.

**FIGURE 2:**
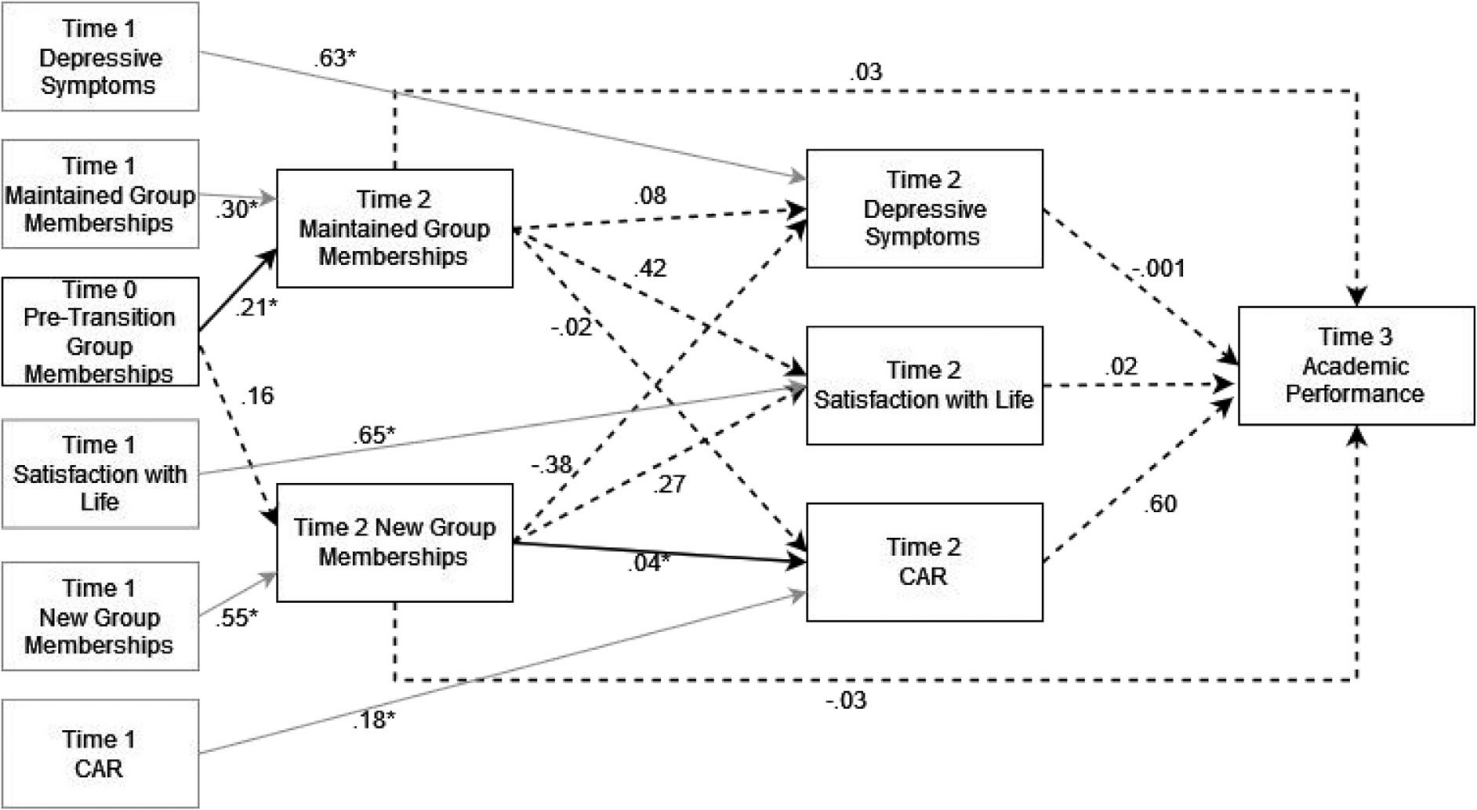
SIMIC pathways predicting health outcomes and academic performance across academic year. Note. *p < .05. Grey boxes and arrows indicate covariates. Dashed arrows indicate non‐significant paths. All T1 variables were allowed to covary. T2 maintained group memberships, and new group memberships were allowed to covary. Health variables at T2 (depression, SWL, CAR) were allowed to covary.

The full model, as displayed in Figure 2, demonstrated good fit, *χ* ^2^(29) = 35.93, *p* = .176; RMSEA = .040 [.0001, .077], CFI = .969, NFI = .881, AIC = 157.93 and AIC_c_ = 241.02.

We hypothesized (H2) that maintained group memberships (T2) would be associated with lower depressive symptoms (T2; *p* = .893), greater life satisfaction (T2; *p* = .153), better academic performance (T3; *p* = .401) and a larger CAR (T2; *p* = .161); this was not supported. H3 was partially supported: New group memberships were associated with a larger CAR, *b* = .035, *p* = .025, indicative of a better ability to cope with stress. However, group membership gain was not associated with depressive symptoms (*p* = .492), life satisfaction (*p* = .338) or academic performance (*p* = .407). Neither social identity continuity nor gain indirectly predicted academic performance via the mediators of depressive symptoms, CAR and life satisfaction (continuity; *b* = −.006, 95% CI [−0.13, 0.11], gain; *b* = .065, [−0.10, 0.24]); H4 was not supported.

## DISCUSSION

The present research explored if SIMIC's social identity continuity and gain pathways could predict CAR (a stress biomarker), well‐being and academic performance. This research presents a novel addition to the literature, demonstrating that social identity gain (i.e. new social group memberships formed after starting university) was associated with greater post‐awakening cortisol concentrations across time. In terms of psychological well‐being, early in the first semester (T1), social membership continuity and gain were related to psychological well‐being, but this was not observed longitudinally, nor did they predict end‐of‐year academic performance.

This research was the first to consider if social identity change would be associated with a stress biomarker during this transitional period, extending on past work which has focused on psychological well‐being and academic performance (Cruwys et al., 2021; Foran et al., 2023; Praharso et al., 2017). Students who joined more social groups across the first few months of university demonstrated larger increases in CAR, indicative of a greater ability to cope with stress (Powell & Schlotz, 2012). This response may be indicative of psychological resilience to stress (Aizpurua‐Perez et al., 2023; O'Connor et al., 2021), as through new social group memberships, these students may have had more psychological resources to draw upon. However, maintenance of group memberships was not associated with CAR. The results provide preliminary evidence that social group membership *gain* is associated with a stress biomarker. Whilst this is only initial evidence, if group membership gain promotes more healthful coping, as reflected in stress biomarkers such as CAR, this may be one mechanism through which group memberships may protect physical health in the longer term (i.e. less stress dysregulation).

The results showed that at time 1 students who reported greater social identity continuity and gain also reported fewer depressive symptoms and more life satisfaction. This is consistent with past research showing that social group memberships buffer the negative effects of stressful transitions (Cruwys et al., 2021; Praharso et al., 2017). However, when we examined this across the semester (T2), there were no associations. Interestingly, while Cruwys et al. (2021) found social identity continuity was important for life satisfaction longitudinally, it did not predict mental health in their sample; and social identity gain predicted neither.

Contrary to our hypotheses, SIMIC processes did not predict academic performance (neither directly norindirectly). We predicted these effects on the basis of previous literature and our rationale that academic performance is a marker of successful transition in this context. However, it may be that academic performance is too distal an outcome and SIMIC is more relevant for health‐ and well‐being‐related outcomes specifically. Alternatively, it may be the case that student or discipline‐specific identities would be more relevant to academic performance than multiple group membership as a global construct.

It is worth noting that the type of student population in this research (students resident in Ireland adjusting to an Irish university) differs from past research which focused on international students (Cruwys et al., 2021; Praharso et al., 2017), which may explain some of the contrasting findings. International students face different challenges compared to domestic students, as they deal with additional adjustments in adapting to a new country with distinct cultural norms, societal expectations and languages. This could potentially make the support and stability provided by maintained groups more protective in this cohort, whereas domestic students, like those in our sample, have the advantage of being able to return home at the weekends, stay in familiar environments and reconnect with pre‐university groups.

Overall, considering the theoretical implications of SIMIC, we find some support that social identities influence well‐being during transitions — at least in the early stages of university. Notably, this research presents an important addition to this literature by showing how SIMIC's social identity gain pathway relates to more adaptive post‐awakening cortisol concentrations across time. Our findings carry policy implications for higher education institutions globally, many of which are facing challenges with student retention, with students often reporting poor health during this transition period (Cage et al., 2021). We suggest higher education institutions consider the implications of SIMIC's social identity pathways demonstrated in this paper. This could involve prioritising the development and implementation of programmes such as academic cohort programs, learning communities or social groups and organisations designed to cultivate connections among students who share common interests and identities (Miller & Vaughn, 2023; Morosanu et al., 2010; Tinto, 2017). Indeed, social group interventions such as Groups4Health have been shown to build social connectedness and reduce depressive symptoms across a variety of settings (Cruwys et al., 2022; Haslam et al., 2019) and could be implemented by universities. Further, peer‐led social support interventions for first‐year students have shown positive effects on social adjustment during this transition period (Mattanah et al., 2010). Whilst in its early stages, this research provides initial evidence that social identity gain influences a stress biomarker in the body, which may over time influence physical health.

### Limitations

Although the present research contributes valuable insights into the relationships between social group memberships and health, several limitations should be acknowledged. Due to attrition, there was a relatively small sample size at T2 and for CAR analyses. As such, we only had statistical power to detect medium‐to‐large effects, despite the use of FIML, and the results should be interpreted with this in mind. It is also worth noting that path analysis is limited in handling measurement errors relating to latent constructs, which may have led to less accurate estimates compared to a full SEM (Bollen & Lennox, 1991; Bollen & Pearl, 2013). Furthermore, participants are from a specific geographic area which may affect the generalizability of the results to a more diverse student population. Whilst a larger increase in CAR may be indicative of psychological resilience, as this was not measured directly, conclusions in this regard are limited. Assessment of resilience in this context is an avenue for future research. Future research could also employ a range of health biomarkers to build on the observed findings, for example assessment of oxytocin which is an important marker in social bonding (e.g. Inagaki et al., 2020). Nevertheless, our study may set a new path in research in this area, indicating potential differences in the benefits of group memberships for domestic and international students.

## CONFLICT OF INTEREST STATEMENT

The authors declare no conflicts of interest.

## ETHICS STATEMENT

Ethical approval was obtained from the institutional ethics board (2021_09_01_EHS). All data is available open access on the Open Science Framework (link provided in the “Methods” section).

## Supporting information


**Data S1.** Supporting Information.

## Data Availability

All data are available on the Open Science Framework: https://osf.io/5xabk/?view_only=bdbae4f6ae674d6db29f117fd6b9b7a4.

## ENDNOTES

1 To assess the robustness of the SIMIC‐derived model, we evaluated the fit of this model compared to four alternative models; see Supplementary Materials.

2 Data available at: https://osf.io/5xabk/?view_only=bdbae4f6ae674d6db29f117fd6b9b7a4.

3 A series of *t*‐tests were conducted to test for differences between participants who completed all elements of the study or not; see Supplementary Materials and Table S3.
