# Supplementary material for: Identity change and the transition to university: Implications for cortisol awakening response, psychological well‐being and academic performance
Source: Appl Psychol Health Well Being. 2024 Oct 18;17(1):e12608. doi: 10.1111/aphw.12608 (PMC11635921; doi:10.1111/aphw.12608)
Supplement: Supplementary file 1 — Data S1. Supporting Information. [file APHW-17-0-s001.docx]

**Table S1**

*Demographics & Means (and SDs) at Each Time-point, and for Those Who Opted into Cortisol Assessment*

|  | Full Sample | | |  | Cortisol Subsample | | |
| --- | --- | --- | --- | --- | --- | --- | --- |
|  | T1 (*N* = 153) |  | T2 (*N* = 79) |  | T1 (*N* = 58) |  | T2 (*N* = 38) |
| Age | 18.52 (0.61) |  | 18.46 (0.50) |  | 18.45 (0.56) |  | 18.41 (0.50) |
| Gender (*n*) |  |  |  |  |  |  |  |
| Female | 106 |  | 57 |  | 42 |  | 29 |
| Male | 44 |  | 19 |  | 16 |  | 9 |
| Gender Fluid | 1 |  | 1 |  | - |  | - |
| Other/not disclosed | 2 |  | 2 |  | - |  | - |
| CAR | 4.14 (7.92) |  | 2.17 (1.72) |  | 4.14 (7.92) |  | 2.17 (1.72) |
| Log CAR | 0.54 (0.31) |  | 0.46 (0.18) |  | 0.54 (0.31) |  | 0.46 (0.18) |
| Depressive symptoms | 12.97 (9.43) |  | 12.63 (9.57) |  | 12.75 (10.26) |  | 10.88 (8.43) |
| SWL | 16.51 (4.42) |  | 16.41 (4.93) |  | 16.55 (4.51) |  | 16.05 (5.03) |
| Groups Pre-university | 4.10 (1.54) |  | - |  | 3.96 (1.58) |  | - |
| Groups Maintained | 4.13 (1.56) |  | 4.29 (1.49) |  | 3.77 (1.41) |  | 4.01 (1.52) |
| New Groups | 4.27 (1.68) |  | 4.79 (1.56) |  | 4.41 (1.74) |  | 4.87 (1.61) |

**Figure S1**

*Study Flowchart of Participant Participation, Analysis Numbers, and Attrition Rates*


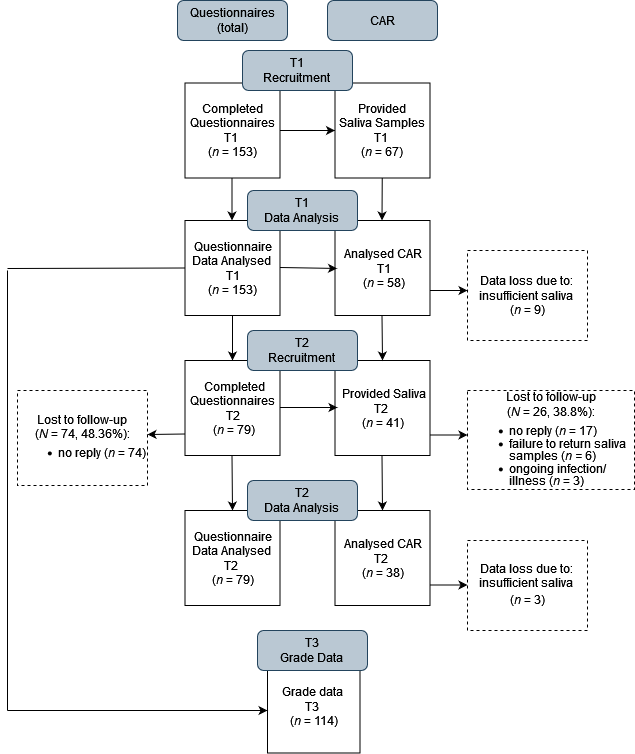


**Participant Restrictions.** Participants opting into CAR assessment could only provide saliva if they reported good health (i.e., free from colds/flus), were not taking medications such as glucocorticoids or immunosuppressants, did not have a chronic immune condition (e.g., diabetes, arthritis), a heart-related illness (e.g., angina, high blood pressure) or oral/periodontal disease, were non-smokers, and not pregnant; as these factors are known to influence the CAR. Individuals who reported colds/flu were invited to take part in Week 4 (instead of Week 3) if they felt fully recovered.

**Materials & Apparatus**

**Table S2**

*Cronbach’s Alphas for each Psychometric Measure, at Each Time-point*

| Measures | Time 1 |  | Time 2 |
| --- | --- | --- | --- |
| EXITS |  |  |  |
| Groups Pre-University | .892 |  | - |
| Groups Maintained | .869 |  | .837 |
| New Groups | .903 |  | .876 |
| BDI-II | .906 |  | .917 |
| SWL | .805 |  | .865 |

*Note.* BDI-II = Beck Depression Inventory-II, SWL = Satisfaction with Life Scale.

**Supplementary Results**

**Preliminary Analyses**

There were no differences between participants who completed the measures at both time-points vs. T1 only completers on any psychometric measure or on CAR, all *p*s > .105 (see Table S3). In terms of participants who opted in/out of the cortisol element, aside from one significant difference where students who opted into the cortisol element of the study at T1 (*n* = 67) reported significantly lower group maintenance at T1 (*M* = 3.77, *SD* = 1.41) compared to students who only completed the psychometric measures only (*n* = 86; *M* = 4.41, *SD* = 1.61), *t*(151) = 2.57, *p* = .011, 95% CI [-1.13, -0.15], there were no other significant differences, all *p*s >.309 (see Table S3). Further, at T2 there was no significant difference between these groups on group maintenance, *p* = .089.

**Table S3**

*Examination of Differences between Individuals Who Completed Psychometric Measures at Both Time-points (n = 79), and Just the First Time-point (n = 74)*

|  | *t* | *df* | *P* | Lower CI | Upper CI |
| --- | --- | --- | --- | --- | --- |
| CAR | 0.75 | 56 | .455 | -3.24 | 7.14 |
| CAR Log | 0.59 | 56 | .555 | -0.14 | 0.23 |
| Depressive symptoms | 0.16 | 151 | .877 | -2.79 | 3.26 |
| SWL | 0.84 | 150 | .401 | -0.81 | 2.02 |
| Groups Pre-University | 1.63 | 149.42 | .105 | -0.89 | 0.85 |
| Maintained Groups | 0.82 | 139.24 | .414 | -0.71 | 0.29 |
| New Groups | 1.19 | 151 | .237 | -0.21 | 0.86 |

**Table S4**

*Examination of Differences Between Individuals Who Opted into the Cortisol Assessment or Opted Out, at Both Time-points*

|  | Time 1 | | | | |  | Time 2 | | | | |
| --- | --- | --- | --- | --- | --- | --- | --- | --- | --- | --- | --- |
|  | *t* | *df* | *P* | Lower CI | Upper CI |  | *t* | *df* | *p* | Lower CI | Upper CI |
| Depressive symptoms | 0.26 | 151 | .793 | -3.45 | 2.64 |  | 1.67 | 77 | .099 | -7.80 | 0.68 |
| SWL | 0.79 | 150 | .937 | -1.38 | 1.49 |  | -0.67 | 74 | .508 | -3.02 | 1.51 |
| Groups Pre-University | 1.02 | 151 | .309 | -0.75 | 0.2 |  | - | - | - | - | - |
| Maintained Groups | 2.57 | 151 | .011* | -1.13 | 0.15 |  | -1.72 | 77 | .089 | -1.23 | 0.89 |
| New Groups | 0.88 | 151 | .379 | -0.30 | 0.78 |  | 0.46 | 77 | .644 | -0.54 | 0.87 |

*Note.* *Indicates a significant difference

**Table S5**

*Bivariate Correlations Between All Variables*

|  | 2 | 3 | 4 | 5 | 6 | 7 | 8 | 9 | 10 | 11 | 12 |
| --- | --- | --- | --- | --- | --- | --- | --- | --- | --- | --- | --- |
| 1. Groups Pre- University (T0) | +.166* | +.079 | +.059 | +.103 | +.213 | +.279* | +.163 | +.143 | -.101 | -.012 | -.026 |
| 2. Groups Maintained (T1) |  | -.004 | -.264** | +.242** | +.069 | +.317** | +.101 | -.039 | +.219 | +.033 | +.144 |
| 3. Groups New (T1) |  |  | -.193* | +.263** | -.005 | +.141 | +.600** | -.166 | +.129 | +.247 | +.029 |
| 4. Depression (T1) |  |  |  | -.571** | -.079 | -.130 | -.115 | +.611** | -.413** | -.204 | -.151 |
| 5. SWL (T1) |  |  |  |  | -.003 | +.230* | +.098 | -.443** | +.624** | +.094 | +.085 |
| 6. CAR (T1) |  |  |  |  |  | +.076 | +.303* | -.280 | +.267 | +.377* | +.051 |
| 7. Groups Maintained (T2) |  |  |  |  |  |  | +.235* | -.081 | +.259* | -.037 | +.091 |
| 8. Groups New (T2) |  |  |  |  |  |  |  | -.127 | +.171 | +.385* | +.013 |
| 9. Depression (T2) |  |  |  |  |  |  |  |  | -.552** | +.008 | -.029 |
| 10. SWL (T2) |  |  |  |  |  |  |  |  |  | -.142 | +.178 |
| 11. CAR (T2) |  |  |  |  |  |  |  |  |  |  | +.112 |
| 12. Academic Performance (T3) |  |  |  |  |  |  |  |  |  |  |  |

*Note.* SWL = Life Satisfaction, CAR = Cortisol Awakening Response. **p* < 05, ***p* < .01

**Model Fit Statistics for Alternative Models**

We also evaluated the fit of the theoretical model presented in the main manuscript compared to four alternative possible models, to assess the robustness of the SIMIC-derived model. Consistent with past research (see Cruwys et al., 2021), we tested: (i) a strict chronological model where each T1 variable was allowed to predict only its T2 counterpart; (ii) a full cross-lagged model, where all T1 variables were allowed to predict all T2 variables, which in turn predicted academic performance (T3); (iii) an inverted model, where T2 variables of depressive symptoms, CAR, and life satisfaction were allowed to predict T2 social identity change variables (new and maintained groups); and (iv) a baseline group variables model, where only T1 measures of group membership were included as predictors.

As can be seen in Table S6, examination of the fit indices suggests that our theoretically derived model was the best fit to the data compared to the other models tested. Full results for the other models are reported in Figures S2-S5.

**Table S6**

*Fit Statistics for the Tested Models*

| Model | χ^2^(df) | *P* | CFI | NFI | RMSEA | AIC | AIC_c_ |
| --- | --- | --- | --- | --- | --- | --- | --- |
| **1. Theoretical-derived model** | **35.93(29)** | **.176** | **.969** | **.881** | **.040[.0001, .077]** | **157.93** | **241.02** |
| 2. Chronological model | 76.51(41) | .001 | .841 | .746 | .075[.049, .101] | 174.51 | 222.08 |
| 3. Full cross-lagged model | 33.60(16) | .006 | .921 | .889 | .085[.044, .125] | 181.60 | 323.91 |
| 4. Inverted model | 62.87(31) | .001 | .857 | .792 | .082[.053, .111] | 180.87 | 257.00 |
| 5. Baseline group variables model | 62.87(23) | <.001 | .764 | .719 | .107 [.076, .139] | 146.87 | 179.71 |

*Note.* Numbers in bold = best fitting model

**Figure S2**

*Strict Chronological Model*

**
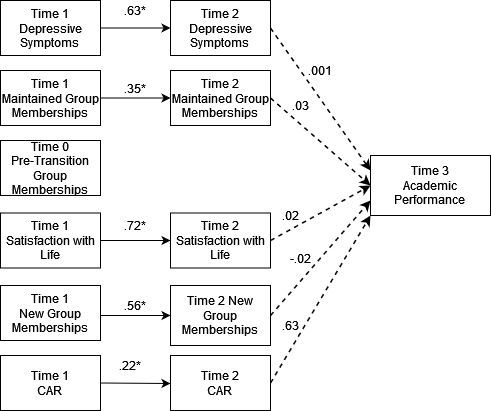
**

*Note.* All T1 variables were allowed to covary, and uncorrelated error terms were specified for all the endogenous variables. Dashed arrows indicate non-significant paths. * *p* < .05

**Figure S3**

*Fully Cross-Lagged Model*

**
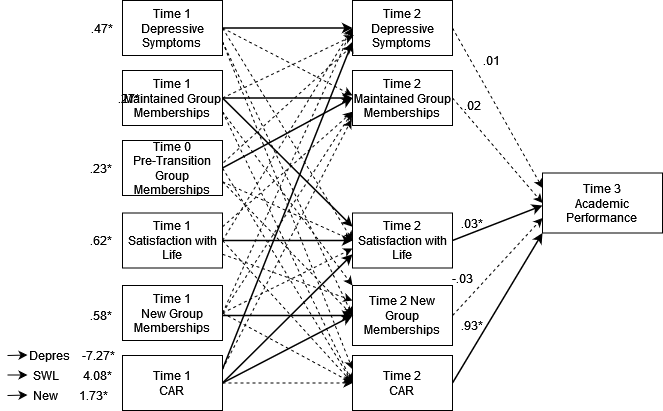
**

*Note.* All T1 variables were allowed to covary, and uncorrelated error terms were specified for all the endogenous variables. Dashed arrows indicate non-significant paths. Due to space constricts standardised coefficients are provided for significant paths on the left of the figure. * *p* < .05

**Figure S4**

*Inverted Model*

**
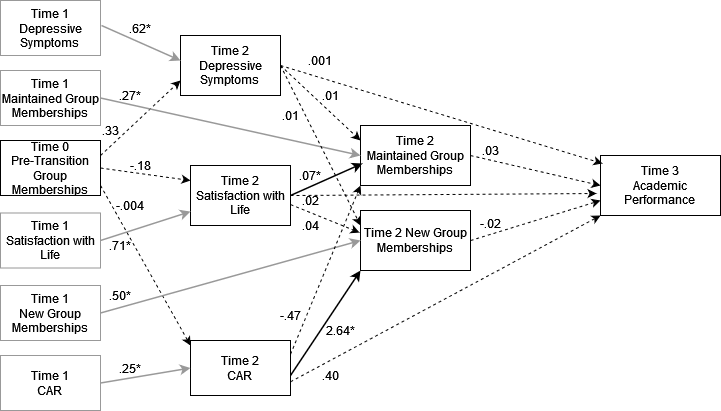
**

*Note.* All T1 variables were allowed to covary, and uncorrelated error terms were specified for all the endogenous variables. Dashed arrows indicate non-significant paths. T2 maintained group memberships and new group memberships were allowed to covary. * *p* < .05

**Figure S5**

*Baseline Model*

**
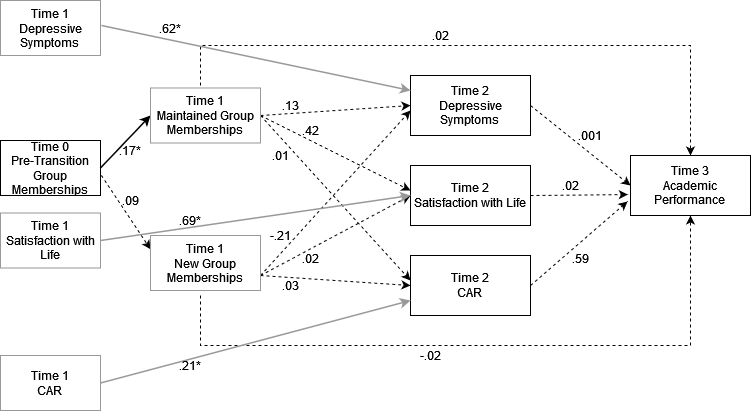
**

*Note.* All T1 variables were allowed to covary, and uncorrelated error terms were specified for all the endogenous variables. Dashed arrows indicate non-significant paths. * *p* < .05

**Model Fit Statistics for Full SEM with Latent Constructs**

For interest, we report model fit statistics and regression paths for a full SEM with latent constructs for the SIMIC theoretical model. Due to missing data points, multiple imputation in AMOS was used (regression imputation). We therefore advise caution in interpreting these results as data imputation with a large proportion of missing data is not advised (Lee & Huber, 2021).

The full model, as displayed in Figure S6, demonstrated adequate fit, χ^2^(2311) = 4560.81, *p* < .001; RMSEA = .080 [.077, .083], CFI = .714, NFI = .556, AIC = 5048.81, AIC_c_ = 1354.44.

For clarity, pathways between each scale item and their respective latent variable are not shown in Figure S6. For the T1 latent variables of depression, life satisfaction, new group memberships, and maintained group memberships, all individual scale items regressed significantly onto their latent constructs, all *p*s < .007. For the T2 latent variables of life satisfaction, new group memberships, and maintained group memberships, all individual scale items regressed significantly onto their latent constructs, all *p*s < .001. Similarly, all BDI-II items at T2 with the exception of item 18 (relating to weight loss, *p* = .053) regressed onto the latent construct of depression (all *p*s < .001).

In this model, with imputed data, maintained group memberships (T2) were not significantly associated depressive symptoms (T2; *p* = .442). However, greater maintenance of group memberships was associated with greater life satisfaction (T2; *b* = .127, *p* = .006), a lower increase in post-awakening cortisol responding (T2; *b* = -.035, *p* < .001), and greater academic performance (T3; *b* = .078, *p* = .028). As such H2 was partially supported. People who reported maintaining more social group memberships reported greater life satisfaction, and also performed better academically. Contrary to hypotheses, this was also associated with a lower CAR. However, results need to be interpreted with caution due to data imputation for many CAR values.

We hypothesized (H3) that new group memberships (T2) would be associated with lower depression (T2), greater life satisfaction (T2), a larger CAR (T2), and greater academic performance (T3). This was partially supported: new group memberships were associated with a larger CAR, *b* = .045, *p* < .001, indicative of an adaptive response profile. However, group membership gain was not associated with depressive symptoms (*p* = .190), life satisfaction (*p* = .952), or academic performance (*p* = .064).

Further, depressive symptoms (*p* = .864) and life satisfaction (*p* = .121) were not directly associated with academic performance. However, greater increases in CAR were associated with greater academic performance (*b* = 1.05, *p* = .007).

Social identity gain was indirectly associated with academic performance, *b* = .145, [0.01, 0.10]. It appears that new social group memberships were associated with greater CAR which in turn was associated with better academic performance; partially supporting H4. However, academic performance was not indirectly predicted by social identity continuity via the mediators of depressive symptoms, CAR, and life satisfaction (*b* = -.058, 95% CI [-0.06, 0.01]). Again, due to the use of multiple imputation, these results should be interpreted with caution.

**Figure S6**

*SEM Model with Latent Variables, Using Data Imputation*


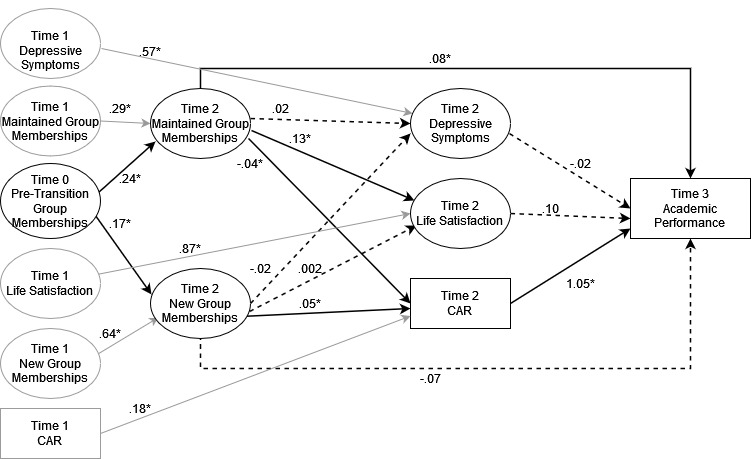


*Note.* **p* < .05. Oval boxes indicate latent variables. Grey boxes and arrows indicate covariates. Dashed arrows indicate non-significant paths. All T1 variables were allowed to covary. T2 maintained group memberships and new group memberships were allowed to covary. Health variables at T2 (depression, SWL, CAR) were allowed to covary.

**References**

Cruwys, T., Ng, N. W. K., Haslam, S. A., & Haslam, C. (2021). Identity Continuity Protects Academic Performance, Retention, and Life Satisfaction among International Students. *Applied Psychology*, *70*(3), 931–954. https://doi.org/10.1111/apps.12254

Lee, J. H., & Huber, J. C. (2021). Evaluation of Multiple Imputation with Large Proportions of Missing Data: How Much Is Too Much? *Iranian Journal of Public Health*, *50*(7), 1372–1380. https://doi.org/10.18502/ijph.v50i7.6626

Moshagen, M., & Erdfelder, E. (2016). A New Strategy for Testing Structural Equation Models. *Structural Equation Modeling: A Multidisciplinary Journal*, *23*(1), 54–60. https://doi.org/10.1080/10705511.2014.950896

Praharso, N. F., Tear, M. J., & Cruwys, T. (2017). Stressful life transitions and wellbeing: A comparison of the stress buffering hypothesis and the social identity model of identity change. *Psychiatry Research*, *247*, 265–275. https://doi.org/10.1016/j.psychres.2016.11.039

R Core Team. (2021). *R: A Language and Environment for Statistical Computing.* [R Foundation for Statistical Computing.].

Stalder, T., Kirschbaum, C., Kudielka, B. M., Adam, E. K., Pruessner, J. C., Wüst, S., Dockray, S., Smyth, N., Evans, P., Hellhammer, D. H., Miller, R., Wetherell, M. A., Lupien, S. J., & Clow, A. (2016). Assessment of the cortisol awakening response: Expert consensus guidelines. *Psychoneuroendocrinology*, *63*, 414–432. https://doi.org/10.1016/j.psyneuen.2015.10.010

Stalder, T., Lupien, S. J., Kudielka, B. M., Adam, E. K., Pruessner, J. C., Wüst, S., Dockray, S., Smyth, N., Evans, P., Kirschbaum, C., Miller, R., Wetherell, M. A., Finke, J. B., Klucken, T., & Clow, A. (2022). Evaluation and update of the expert consensus guidelines for the assessment of the cortisol awakening response (CAR). *Psychoneuroendocrinology*, *146*, 105946. https://doi.org/10.1016/j.psyneuen.2022.105946
